# Endoscopic imaging modalities for diagnosing invasion depth of superficial esophageal squamous cell carcinoma: a systematic review and meta-analysis

**DOI:** 10.1186/s12876-017-0574-0

**Published:** 2017-02-02

**Authors:** Ryu Ishihara, Noriko Matsuura, Noboru Hanaoka, Sachiko Yamamoto, Tomofumi Akasaka, Yoji Takeuchi, Koji Higashino, Noriya Uedo, Hiroyasu Iishi

**Affiliations:** Department of Gastrointestinal Oncology, Osaka Medical Center for Cancer and Cardiovascular Diseases, 1-3-3 Nakamichi Higashinari-ku, Osaka, 537-8511 Japan

**Keywords:** Esophageal cancer, Cancer invasion depth, Endoscopy, Magnified endoscopy, Endosonography, Squamous cell carcinoma

## Abstract

**Background:**

Diagnosis of cancer invasion depth is crucial for selecting the optimal treatment strategy in patients with gastrointestinal cancers. We conducted a meta-analysis to determine the utilities of different endoscopic modalities for diagnosing invasion depth of esophageal squamous cell carcinoma (SCC).

**Methods:**

We conducted a comprehensive search of MEDLINE, Cochrane Central, and Ichushi databases to identify studies evaluating the use of endoscopic modalities for diagnosing invasion depth of superficial esophageal SCC. We excluded case reports, review articles, and studies in which the total number of patients or lesions was <10.

**Results:**

Fourteen studies fulfilled our criteria. Summary receiver operating characteristic curves showed that magnified endoscopy (ME) and endoscopic ultrasonography (EUS) performed better than non-ME. ME was associated with high sensitivity and a very low (0.08) negative likelihood ratio (NLR), while EUS had high specificity and a very high (17.6) positive likelihood ratio (PLR) for the diagnosis of epithelial or lamina propria cancers. NLR <0.1 provided strong evidence to rule out disease, and PLR >10 provided strong evidence of a positive diagnosis.

**Conclusions:**

EUS and ME perform better than non-ME for diagnosing invasion depth in SCC. ME has a low NLR and is a reliable modality for confirming deep invasion of cancer, while EUS has a high PLR and can reliably confirm that the cancer is limited to the surface. Effective use of these two modalities should be considered in patients with SCC.

**Trial registration:**

PROSPERO (International Prospective Register of Systematic Reviews); number 42015024462.

## Background

Esophageal squamous cell carcinoma (SCC) is one of the common causes of cancer-related mortality worldwide [1]. Although the overall survival of patients with esophageal SCC remains poor, it can potentially be cured by esophagectomy, endoscopic resection (ER) or chemoradiotherapy if diagnosed at an early stage [2–7]. Esophagectomy has been the mainstay of treatment for superficial esophageal SCC. However, this procedure is only possible in patients able to tolerate the procedure, and is associated with significant mortality and substantial morbidity [8, 9]. Endoscopic therapy offers an alternative, minimally invasive option for patients with superficial esophageal SCC. Although both these treatments are applicable for superficial esophageal SCC, they differ greatly in terms of their invasiveness.

Many factors, e.g. the patient’s condition, metastatic status, cancer invasion depth, and size of the lesion, must be taken into account when choosing the appropriate treatment. Among these factors, cancer invasion depth correlates well with the risk of metastasis and the curability by ER [10, 11]. Diagnosis of cancer invasion depth is therefore crucial for selecting the optimal treatment strategy in patients with esophageal SCC.

Many modalities, e.g. non-magnified endoscopy (non-ME), magnified endoscopy (ME), and endoscopic ultrasound (EUS) are currently used for diagnosing the invasion depth of superficial esophageal SCC. Non-ME is a conventional diagnostic modality for invasion depth, and the diagnosis is usually based on the protrusion, depression, thickness, and hardness of the esophageal wall. However, diagnosis by non-ME is subjective and may be subject to inter-observer variability. ME allows clear observation of the microvascular architecture, which is closely associated with the development of esophageal cancer. Diagnosis of esophageal cancer invasion depth using ME was introduced in the 1990s [12, 13]. This modality requires image-enhancement and magnifying functions, but can lead to a rapid and objective diagnosis. EUS is the most popular of the three modalities, but has produced conflicting results [14, 15] regarding its utility for diagnosing superficial esophageal SCC. There is thus currently no consensus on the best modality for diagnosing invasion depth in patients with superficial esophageal SCC. We therefore conducted a meta-analysis to elucidate the utilities of these modalities for the diagnosis of esophageal cancer invasion depth.

## Methods

### Search strategy

We searched the MEDLINE, Cochrane Central, and Ichushi databases from January 1995 to June 2015 using the following search terms: (“esophageal cancer” OR “esophageal tumor” OR “esophageal tumor” OR “esophageal neoplasia” OR “esophageal carcinoma” OR “esophageal mucosal” OR “esophageal lamina propria”) AND (“diagnosis” OR “endosonography” OR “staining and labeling” OR “iodine” OR “magnifying endoscopy OR “chromoendoscopy” OR “NBI” OR “avascular area” OR “endoscopic ultrasound” OR “imaging” OR “pathology” OR “esophagoscopy”) AND (“neoplasm invasiveness” OR “[T1a and EP]” OR “M1” OR “Tis” OR “[T1a and LPM]” OR “M2” OR “T1a” OR “(T1a and MM)” OR “M3” OR “T1b” OR “[pT1a and MM]” OR “T1b” OR “SM” OR “SM1” OR “SM2” OR “SM3” OR “[T1b and SM] OR “vascular involvement” OR invasion OR “infiltration” OR “depth”). Our search was restricted to English- or Japanese-language studies of human subjects. Two reviewers (R.I. and N.M.) independently screened the titles and abstracts of all the articles according to the defined inclusion and exclusion criteria. The final complete report of all selected articles was then retrieved and reviewed by the same two reviewers (R.I. and N.M.). We also manually screened the reference lists of the selected articles for any potential related articles that were not identified by the initial search (Manual searching). Discrepancies were resolved by discussions. The protocol for this meta-analysis was registered in PROSPERO (International Prospective Register of Systematic Reviews; number 42015024462), in accordance with the most recently published guidelines [16].

### Inclusion and exclusion criteria

The study population consisted of patients with esophageal SCC based on endoscopic biopsy and endoscopic examination. The intervention was endoscopic diagnosis (non-ME, ME or EUS) of cancer invasion depth for superficial SCC. The reference standard was histologic diagnosis of cancer invasion depth by ER, or from surgically resected specimens. Acceptable study designs were retrospective or prospective studies with sufficient data to allow reconstruction of a diagnostic 2 × 2 table (true positive, false positive, true negative, and false negative). We excluded case reports, review articles, and studies in which the total number of patients or lesions was <10. We also excluded studies that did not provide any predefined criteria to diagnose invasion depth and studies with imaging modalities that are not used in daily practice.

### Cancer invasion depth

Histologic diagnosis of cancer invasion depth was divided into six categories, based on the findings: EP (cancer limited to the epithelium); LPM (cancer invading into the lamina propria); MM (cancer invading into the muscularis mucosa); SM1 (cancer invading 0.2 mm below the lower border of the muscularis mucosa in endoscopically resected specimens and cancer invading the upper third of the submucosal layer in surgically resected specimens); SM2 (cancer invading >0.2 mm into the submucosa in endoscopically resected specimens and cancer invading the middle third of the submucosal layer in surgically resected specimens); SM3 (cancer invading the lower third of the submucosal layer in surgically resected specimens) [17].

Endoscopic diagnosis of cancer invasion depth was divided into three categories: EP/LPM, MM/SM1, and ≥ SM2, because these categories correspond well with the risk of metastasis [10] and indication of ER. Moreover, most diagnostic criteria for cancer invasion depth of esophageal SCC were developed to differentiate these three categories, and there are currently no popular non-ME or ME criteria for differentiating between mucosal and submucosal cancers.

### Data abstraction

Two independent reviewers (R.I. and N.M) extracted the following data from the selected studies and added them to standardized data forms: design; country; year of publication; setting; sample size; reference standard; operating frequencies of endoscope and/or probe; number of endoscopic imaging modalities used; and numbers of true-positive, true-negative, false-positive and false-negative values.

Study quality and potential bias were assessed according to the Quality Assessment of Diagnostic Accuracy Studies-2 (QUADAS-2) tool [18], which included four key domains: patient selection, index test, reference standard, and flow timing. Each domain was assessed for risk of bias, and the first three domains were also assessed regarding applicability. Quality assessment of the studies was performed independently by R.I. and N.M, and any disagreement was resolved by discussion.

### Statistical analysis

We constructed 2 × 2 tables for EP/LPM and ≥ MM, and for EP-SM1 and ≥ SM2 for each study, based on comparisons between the endoscopic diagnosis and final histologic diagnosis by ER or esophagectomy. The true-positive, false-positive, true-negative, and false-negative values were then calculated based on the 2 × 2 tables. A summary receiver operating characteristic curve (SROC) was constructed [19]. A SROC is similar to a standard ROC, except that the SROC data are obtained from the sensitivity and specificity values in the individual studies in the meta-analysis. The area under the curve (AUC) of a SROC is an indicator of the performance of a diagnostic modality [19]. A preferred test has an AUC close to 1, and a poor test has an AUC close to 0.5 [20]. The Q* index is the point where the sensitivity and specificity are equal, which is the point closest to the ideal top-left corner of the SROC space [19].

The pooled sensitivity, specificity, PLR, NLR, and diagnostic odds ratio were estimated using a fixed-effect model (Mantel–Haenszel method). Forest plots were used to show the effect size of each study. Heterogeneity was assessed using Cochran’s Q test and the I^2^ measure of inconsistency [21–23]. The Cochran Q test detects heterogeneity by testing the null hypothesis that all studies in a meta-analysis have the same underlying magnitude of effect. Because this test is underpowered to detect moderate degrees of heterogeneity, a *P* value of <0.10 was considered suggestive of significant heterogeneity [24]. The I^2^ index describes the percentage of total variation among studies attributed to heterogeneity rather than chance. A value of 0% indicates no observed heterogeneity, and larger values show increasing heterogeneity. Higgins et al. [21] suggested that I^2^ indexes of 25%, 50%, and 75% represented low, moderate, and high heterogeneity, respectively. For all statistical methods, except for Cochran’s Q test, P <0.05 was regarded as significant. Data were analyzed using Meta-Disc (version 1.4) and Review Manager.

## Results

### Literature search

**Fig. 1:**
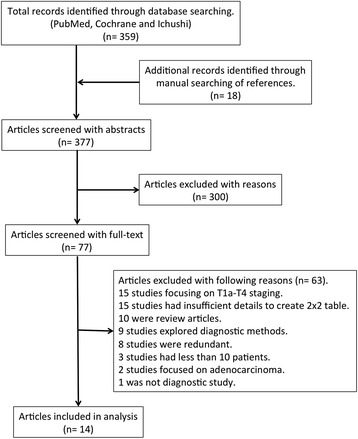
Flow diagram of the study-selection process

A total of 359 articles were initially identified using the search strategy, and 18 additional records were identified through manual searching of references (Fig. 1). Among all the studies, 300 were excluded after preliminary review of the titles and abstracts, leaving 77 articles for detailed evaluation. Of these, 63 articles failed to meet the criteria and 14 studies were finally selected for this meta-analysis [25–38]. Only two of these were prospectively designed studies [27, 36]. All of 14 were Japanese studies and 11 of them were written in Japanese. A total of 359, 1613 and 357 patients received non-ME, ME and EUS, respectively. Details of the studies are described in Table 1 [12, 13, 39].

**Table 1:** Characteristics of included studies

Reference number	Year	Sample size	Modality	Image enhancement	Classification	EUS method	EUS frequencies, MHz	Confirmatory study
25	1997	74	Non-ME	Non	-	-	-	Esophagectomy/ER
26	2010	236	Non-ME	Non	-	-	-	Esophagectomy/ER
27	2015	49	Non-ME/ME	NBI	Arima and Inoue	-	-	Esophagectomy/ER
28	1998	30	ME	Non	Arima	-	-	Esophagectomy/ER
29	2002	79	ME	Non	Inoue	-	-	Esophagectomy/ER
30	2006	12	ME	Non	Inoue	-	-	ER
31	2010	510	ME	FICE	Arima	-	-	Esophagectomy/ER
32	2014	220	ME	NBI	JES	-	-	ER
33	2014	249	ME	NBI	JES	-	-	ER
34	2014	464	ME	NBI	JES	-	-	Esophagectomy/ER
35	1995	40	EUS	-	-	Radial and/or mini-probe	7.5,12,20	Esophagectomy/ER
36	2006	40	EUS	-	-	Mini-probe	20	Esophagectomy/ER
37	2006	132	EUS	-	-	Radial and/or mini-probe	7.5,10,20	Esophagectomy/ER
38	2011	145	EUS	-	-	Mini-probe	20,30	Esophagectomy/ER

*Non-ME* non-magnified endoscopy; *ME* magnified endoscopy; *EUS* endoscopic ultrasound; *IE* Image enhancement method; *FICE* FUJI Intelligent Color Enhancement system; *NBI* Narrow band imaging system; *Arima* Arima’s classification; *Inoue* Inoue’s classification; *JES* Japan esophageal society classification; *ER* endoscopic resection

### Meta-analysis of diagnostic accuracy

**Fig. 2:**
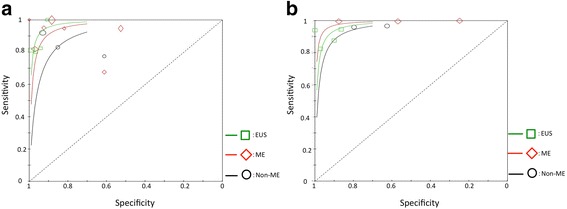
Summary receiver operating characteristic curves for differentiating between EP/LPM and ≥ MM (**a**), and EP-SM1 and ≥ SM2 (**b**). EP: epithelium, LPM: lamina propria, MM: muscularis mucosa, SM: submucosa, EUS: endoscopic ultrasound, ME: magnified endoscopy, Non-ME: non-magnified endoscopy

Summary ROC curves showed that ME and EUS were positioned in the upper right corner of the ROC space compared with non-ME (Fig. 2a, b). The AUC was used to summarize the overall diagnostic accuracy of each modality. Non-ME, ME, and EUS had AUC values of 0.934, 0.946, and 0.975, respectively, for differentiating between EP/LPM and ≥ MM, while ME and EUS had AUC values of 0.999 and 0.966, respectively, for differentiating between EP-SM1 and ≥ SM2.

**Fig. 3:**
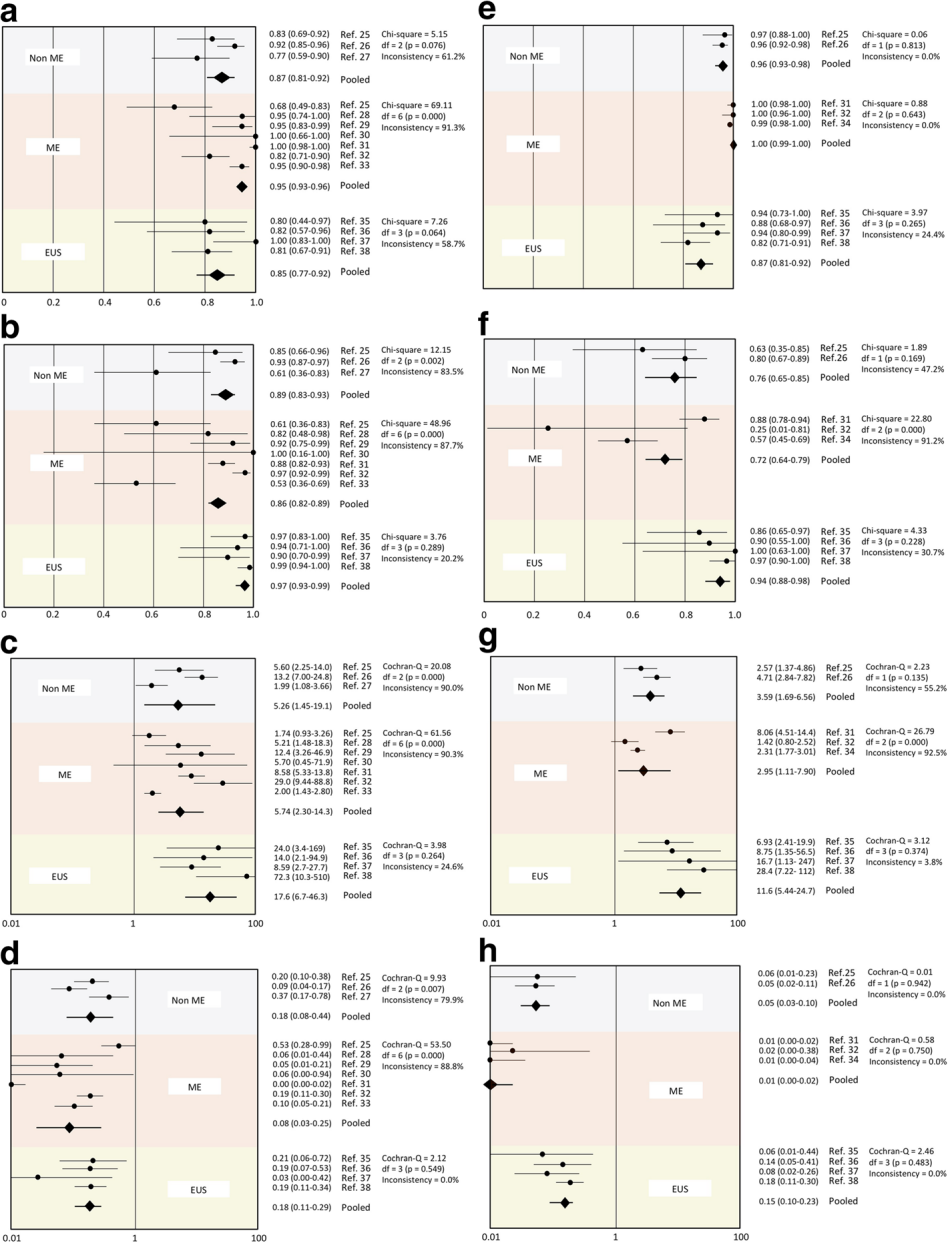
**a** Sensitivity for differentiating between EP/LPM and ≥ MM. **b** Specificity for differentiating between EP/LPM and ≥ MM. **c** Positive likelihood ratio for differentiating between EP/LPM and ≥ MM. **d** Negative likelihood ratio for differentiating between EP/LPM and ≥ MM. **e** Sensitivity for differentiating between EP-SM1 and ≥ SM2. **f** Specificity for differentiating between EP-SM1 and ≥ SM2. **g** Positive likelihood ratio for differentiating between EP-SM1 and ≥ SM2. **h** Negative likelihood ratio for differentiating between EP-SM1 and ≥ SM2. EP: epithelium, LPM: lamina propria, MM: muscularis mucosa, SM: submucosa, EUS: endoscopic ultrasound, ME: magnified endoscopy, Non-ME: non-magnified endoscopy

The Forest plots of sensitivity, specificity, PLR, and NLR for each modality for differentiating between EP/LPM and ≥ MM, and between EP-SM1 and ≥ SM2 are shown in Fig. 3a–d and e–h, respectively. Point estimates with 95% confidence intervals (CIs) were plotted for each group (Fig. 3 a-h). ME had significantly higher sensitivities for diagnosing EP/LPM (0.96 [95%CI: 0.91–0.96]) and EP-SM1 cancers (1.00 [95%CI: 0.99–1.00]) compared with non-ME and EUS. ME also had very low NLR for diagnosing EP/LPM (0.08 [95%CI: 0.03–0.25]) and EP-SM1 cancers (0.01 [95%CI: 0.00–0.02]). EUS showed significantly higher specificities for the diagnosis of EP/LPM (0.97 [95%CI: 0.93–0.99]) and EP-SM1 cancers (0.94 [95%CI: 0.98–0.88]) compared with non-ME and ME. EUS also had a very high PLR for diagnosing EP/LPM (17.63 [95%CI: 6.71–46.34]) and EP-SM1 cancers (11.60 [95%CI: 5.44–24.74]).

### Quality and heterogeneity assessment

**Fig. 4:**
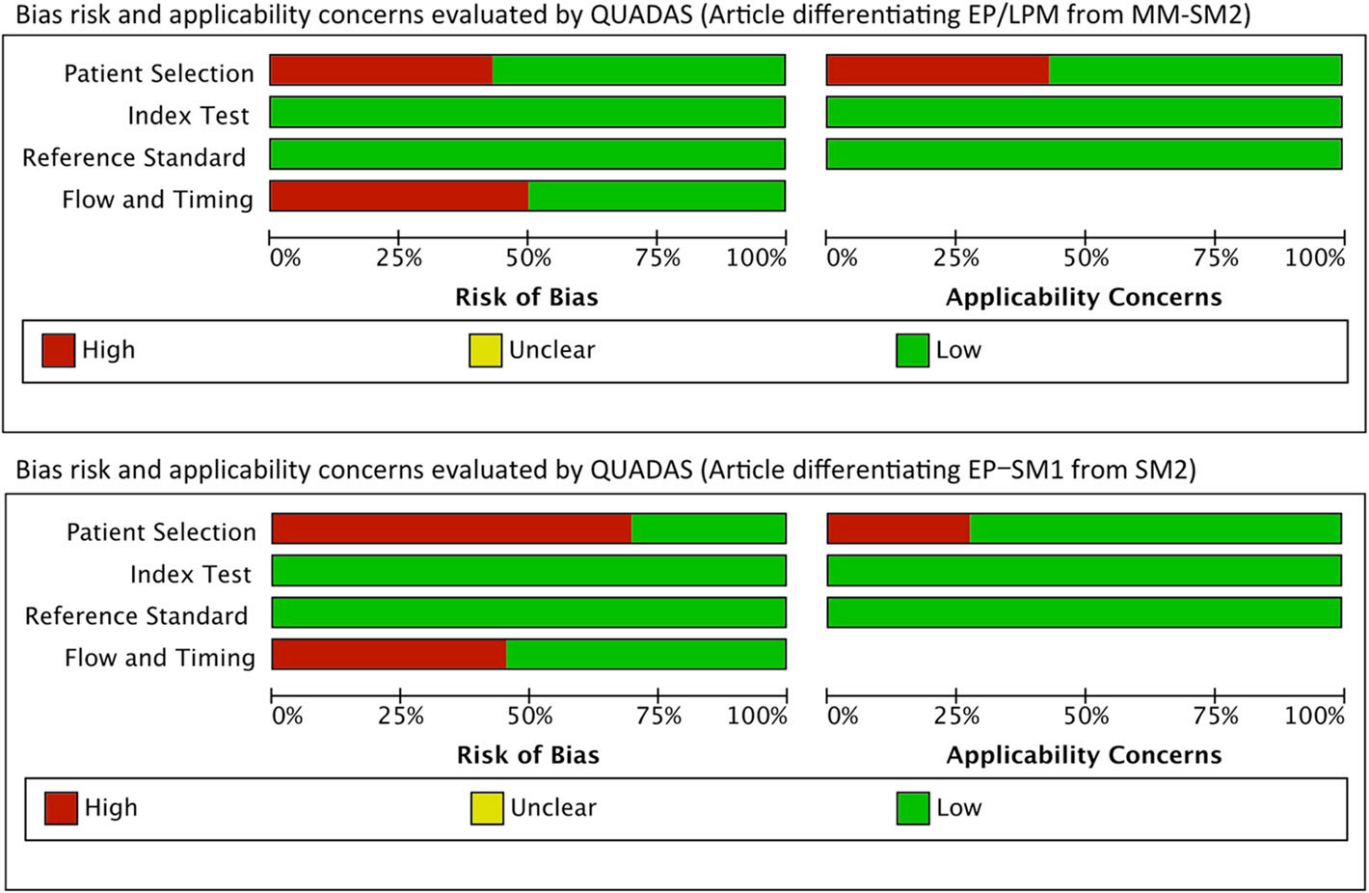
Quality of the included studies evaluated according to the Quality Assessment of Diagnostic Accuracy Studies-2 (QUADAS-2) criteria. EP: epithelium, LPM: lamina propria, MM: muscularis mucosa, SM: submucosa

The qualities of the included studies evaluated according to the QUADAS-2 criteria are shown in Fig. 4. Half the studies showed risk of bias regarding “Patient selection” and “Flow and timing”, mainly as a result of unclear descriptions of the patient-selection process and analysis methods. The Cochran Q test identified heterogeneities for differentiating between EP/LPM and ≥ MM by non-ME (*P* = 0.076 for sensitivity and *P* = 0.002 for specificity) and ME (*P* = 0.002 for sensitivity and *P* < 0.001 for specificity), between EP-SM1 and ≥ SM2 by ME (*P* < 0.001 for specificity). The I^2^ index identified moderate to high heterogeneities for differentiating between EP/LPM and ≥ MM by non-ME (61.2% for sensitivity and 83.5% for specificity) and ME (91.3% for sensitivity and 87.7% for specificity), between EP-SM1 and ≥ SM2 by ME (91.2% for specificity). Sensitivity analysis was not performed because of the limited number of studies of each modality. However, heterogeneity for differentiating between EP/LPM and ≥ MM by non-ME was resolved by excluding one study [27], and heterogeneity for differentiating between EP-SM1 and ≥ SM2 by ME was resolved by excluding another study [31].

## Discussion

The current meta-analysis analyzed the performances of non-ME, ME, and EUS for diagnosing superficial esophageal SCC. SROCs showed a trade-off between sensitivity and specificity. Given that an AUC of 1 indicated an excellent test, EUS and ME demonstrated very high diagnostic accuracies. EUS and ME had different characteristics according to our analysis. ME showed high sensitivities for the diagnosis of EP/LPM and EP-SM1 cancers and very low NLRs for the diagnosis of EP/LPM and EP-SM1 cancers. The NLR assesses the ability of the test to exclude the disease in question. An NLR <0.1 provides strong evidence to rule out the disease [40], indicating that ME is a reliable modality for confirming deep cancer invasion. EUS had high specificities and very high PLRs for the diagnosis of EP/LPM and EP-SM1 cancers. The PLR is a measure of how well the test identified the disease. A PLR >10 provides strong evidence for a positive diagnosis [40], and indicated that EUS was a reliable modality for confirming that the cancer was limited to the surface. Effective use of these two modalities to investigate these characteristics in clinical practice is important.

Although the current meta-analysis analyzed the diagnostic abilities of the individual modalities, they are usually used in combination in clinical practice. Non-ME is conducted as an initial examination in most cases, usually followed by EUS, ME, or both. However, there are currently no guidelines or consensus on how best to combine these modalities, and further studies are therefore needed to clarify the additional benefits of combinations of these modalities.

All the selected articles in the current study were reported from Japan and 11 of them were written in Japanese. This is probably because we limited the disease to SCC, and the cancer invasion depth categories to EP/LPM, MM/SM1 and ≥ SM2. This is one of the limitations of this meta-analysis and may raise some concern about generalizability of the result. This point should be confirmed by further studies outside Japan.

Classification of the invasion depth of superficial esophageal SCCs into three categories (EP/LPM, MM/SM1, and ≥ SM2) is relatively uncommon, but nevertheless practical. It can stratify the risk of metastasis [10], and is therefore closely associated with the indication for ER. According to the Japanese [41] and European [11] guidelines, ER is indicated for EP/LPM cancer, relatively indicated for MM/SM1 cancer, and not indicated for ≥ SM2 cancer. We therefore employed these categories in this meta-analysis.

There were some limitations of this meta-analysis. Non-ME and ME demonstrated heterogeneity for differentiating between EP/LPM and ≥ MM, and ME for differentiating between EP-SM1 and ≥ SM2. We were unable to perform sensitivity analyses because of the limited number of studies for each modality. However, heterogeneities for differentiating between EP/LPM and ≥ MM by non-ME [27], and between EP-SM1 and ≥ SM2 by ME were resolved by excluding one study each [31]. Most of the articles in this meta-analysis were reported from university hospitals or tertiary care hospitals, which specialize in cancer treatment. However, the two studies excluded above were unique; the former was conducted in secondary care general hospitals, and the latter was conducted by one investigator with special expertise in the diagnosis of esophageal SCC [31]. Another limitation of this meta-analysis was the low quality of the studies evaluated by QUADAS-2. Half of the studies had issues of bias regarding “Patient selection” and “Flow and timing”, which may have derived from the patient-selection and analysis processes. These problems cannot be resolved by a retrospective study style, and well-designed prospective studies are required to allow a better meta-analysis to be performed to provide stronger evidence.

## Conclusion

EUS and ME are preferable to non-ME for diagnosing invasion depth in esophageal SCC. ME demonstrated very low NLR, and is thus a reliable modality for confirming deep cancer invasion, while EUS showed a high PLR, and is thus a suitable modality for confirming that a cancer is limited to the surface. Combined use of these two modalities should thus be considered for determining cancer invasion depth in patients with esophageal SCC.

## Abbreviations

AUC: Area under the curve
CI: Confidence interval
EUS: Endoscopic ultrasonography
ME: Magnified endoscopy
NLR: Negative likelihood ratio
PLR: Positive likelihood ratio
QUADAS-2: Quality Assessment of Diagnostic Accuracy Studies-2
SCC: Squamous cell carcinoma
SROC: Summary receiver operating characteristic curve
